# Prospective study of asbestos-related diseases incidence cases in primary health care in an area of Barcelona province

**DOI:** 10.1186/1471-2458-10-203

**Published:** 2010-04-22

**Authors:** Magdalena Rosell-Murphy, Rafael Abós-Herràndiz, Josep Tarrés, Xavier Martínez-Artés, Isabel García-Allas, Ilona Krier, Gloria Cantarell, Miguel Gallego, Ramon Orriols, Constança Albertí

**Affiliations:** 1 IDIAP Jordi Gol. c/Gran Via de Les Corts Catalanes 587 àtic. 08007 Barcelona, Spain; 2 PCT Ripollet.c/Casanovas 4, Ripollet. 08291 Barcelona, Spain; 3 PCT Canaletas. c/Passeig Horta s/n. Cerdanyola del Vallès. 08290 Barcelona, Spain; 4 PCT Serraperera. c/Diagonal s/n. Cerdanyola del Vallès. 08290 Barcelona, Spain; 5 PCT Barberà. Plaça Rosa dels Vents s/n. Barberà del Vallès. 08210 Barcelona, Spain; 6 6. PCT Ripollet.c/Casanovas 4, Ripollet. 08291 Barcelona, Spain; 7 Internal Medicine Department. Corporació Sanitaria Parc Taulí. c/Parc Tauli s/n. 08208 Sabadell, Barcelona, Spain; 8 Respiratory Medicine Department. Corporació Sanitaria Parc Taulí. c/Parc Tauli s/n. 08208 Sabadell, Barcelona, Spain; 9 Respiratory Medicine Department. University Hospital Vall d'Hebron, Psg. Vall d'Hebron, 119-129. 08035 Barcelona. CIBER Respiratory Diseases (CIBERES). Barcelona, Spain; 10 Catalan Institute of Medical Evaluations. Parc sanitari Pere Virgili, Avda Vallcarca 169-205. 08023 Barcelona, Spain

## Abstract

**Background:**

Asbestos related diseases include a number of conditions due to inhalation of asbestos fibres at work, at home or in the environment, such as pleural mesothelioma, asbestosis and calcified pleural plaques. Few epidemiological studies have established the incidence of asbestos related diseases in our area. The present proposal is based on a retrospective study externally funded in 2005 that is currently taking place in the same area and largely carried out by the same research team.

The aim of the study is to achieve a comprehensive and coordinated detection of all new cases of Asbestos Related Diseases presenting to primary care practitioners.

**Methods/design:**

This is a multicentre, multidisciplinary and pluri-institutional prospective study.

Setting

12 municipalities in the Barcelona province within the catchment area of the health facilities that participate in the study.

Sample

This is a population based study, of all patients presenting with diseases caused by asbestos in the study area.

Measurements

A clinical and epidemiological questionnaire will be filled in by the trained researchers after interviewing the patients and examining their clinical reports.

**Discussion:**

Data on the incidence of the different Asbestos Related Diseases in this area will be obtained and the most plausible exposure source and space-time-patient profile will be described. The study will also improve the standardization of patient management, the coordination between health care institutions and the development of preventive activities related with asbestos exposure and disease.

## Background

Humans have used asbestos for centuries. We find the first reference at around 2,500 BC in cremation ceremonies where it is described as a material that burns like wood but does not get burned down when used with oil. The two essential characteristics of asbestos are reflected in this historical note: incombustibility and thermal isolation properties that stand temperatures up to 900°C.

Thanks to these properties, we find asbestos in over 3,000 different applications in most industrial sectors [1,2]. In Barcelona province, the jobs associated with the highest risk are workers at fibrocement plants, painters, carriers of asbestos materials and construction workers [3].

Asbestos is classified into serpentine (curly fibres) and amphibole (straight fibres). Chrysotile or white asbestos belongs to the serpentine group and is the most widespread, amounting to 90% of all industrial asbestos. The chemical structure consists of a silicate compound of different metals (iron, magnesium and calcium) with a fibrous external appearance and a basic physical unit of 50-100 by 0.5-3 μm. It is therefore a true microscopic needle with the ability to enter the aerodynamic structure of the bronchial tree and to reach the smallest and most distal bronchioles and alveoli close to the pleura, thus producing a direct lesion to the lateral and basal lung parenchyma.

Asbestos can also spread via the lymphatic system [2]. Once the asbestos fibres are deposited in the lung, cells are destroyed, fibrosis and perialveolar and bronchiolar scarring takes place (pulmonary asbestosis), ultimately causing breathing difficulty.

Asbestos Related Diseases (ARD) is a group of 10 conditions that affect mainly the respiratory system, caused by the inhalation and subsequent deposit of asbestos fibres in the pulmonary parenchyma. ARD are divided into malignant (pleural mesothelioma, peritoneal mesothelioma, asbestos-related bronchopulmonary carcinoma, and other neoplasms) and non-malignant, in agreement with the guidelines with a broader worldwide consensus [4,5]. The non-malignant ARD comprise diseases of the lung parenchyma (asbestosis or interstitial lung fibrosis), of the pleura (isolated pleural plaques, diffuse pleural fibrosis or pleural thickening and benign pleural effusion), and of the bronchi, with chronic bronchial obstruction and round atelectasis.

Asbestos toxicity is related to its fibrous structure, since it has been shown that pulverized asbestos does not cause disease [6]. Short or low intensity exposure to asbestos does not exclude the possibility of ARD, since there are individual susceptibility factors to asbestosis still poorly understood [7]. The study of risk factors can be hindered by the long latency period between inhalation and clinical disease.

In addition to its fibrogenic properties, asbestos is a first-level carcinogen [2]. The most accepted oncological model is the dose-response without a safety level [8].

The fibrogenic and carcinogenic properties are related, particularly in the amphibole type of asbestos. Its role has been shown in the development of mesothelioma and other pulmonary neoplasms, where it would act as a co-carcinogenic agent [9]. The diagnosis of malignant mesothelioma requires histological and histochemical techniques to differentiate it from metastasis from other cancers. The risk of suffering from a malignant mesothelioma increases with age, and depends on time of exposure. In turn, these factors are related to the level of exposure, continuity of exposure, and latency period [10].

The main risk factor to develop ARD is work exposure, usually in the past, due to inhalation of asbestos fibres. However, not everybody that has been or is in contact with asbestos develops ARD. One of the main difficulties in the study of ARD is the lack of information related to exposure history in the primary care and hospital clinical records of pulmonary fibrosis and lung cancer patients.

This factor, coupled with the late age at diagnosis due to the time gap between exposure and clinical manifestations, explains that a significant number of ARD cases are not considered a work-related disease [10]. Worldwide, a large number of ARD are not considered or notified as a work-related disease [11]. In Spain, asbestosis, asbestos-related lung cancer and mesothelioma are classified as work-related diseases in the Royal Decree 1995/1978 of 12^th ^of May [12]. The scarce notification of these cases as work-related conditions contributes to the lack of knowledge of the real incidence of ARD.

On the other hand, the non-work related exposures that can cause ARD are the consequence of fibre inhalation when living near an asbestos source, living together with an asbestos worker that brought the asbestos fibres in the work clothes, the domestic use of products manufactured with asbestos that are in a poor condition, and finally simple environmental exposure. This last exposure possibility has been studied more deeply in the case of mesothelioma than in asbestos-related lung cancer [13-15]. The incidence of cases of benign ARD, asbestosis comprised, of environmental origin has hardly been approached.

Tobacco consumption is a predisposing factor for ARD. Smokers exposed to asbestos are at higher risk to develop radiologic signs of asbestosis than non-smokers. In turn, asbestosis is a risk factor of lung cancer. Therefore, tobacco consumption would be a non-predisposing concomitant risk factor that has a modulating effect between asbestosis and lung cancer risk [9]. In Spain, around 4% lung cancers cases are related to asbestos work exposure in synergy with tobacco consumption [16]. No association has been found between tobacco consumption and mesothelioma [2].

The clinical presentation of ARD ranges from a subclinical, asymptomatic stage, the casual finding of a dry and persistent cough, to a severe and chronic or acute dyspnoea, in the case of mesothelioma. Expectoration, bronchial obstruction and poor resolution of respiratory symptoms are not pure manifestations of asbestos disease, but of its associated bronchial component. The first descriptive studies carried out 25 years ago in the industrial area of Barcelona indicated that up to 25% of exposed workers could present with ARD [17]. Asbestosis can also be diagnosed when a routine chest X-ray detects irregular, little opacities [9].

The definitive diagnosis of ARD is reached by histology, through the cytological detection of asbestos bodies. In most cases this test is not available and the diagnosis is based on imaging, a history of exposure with an appropriate latency period, and ruling out other possibilities [5].

The imaging techniques can show a diffuse thickening of the pleura (mainly visceral), pleural plaques (mainly parietal, calcified or not calcified), massive or partial pleural effusion, pleural masses and even a bibasilar honeycombing pattern, characteristic of pulmonary fibrosis [6]. The high-resolution CT scan is more accurate and sensitive than conventional X-ray and conventional CT scan, which has a low predictive value of 50%.

The only method of ARD primary prevention is to avoid the inhalation of asbestos fibres. To achieve this, the industry that manipulates this product is subjected to strict safety regulations and constant controls to eliminate every possibility of respiratory or topical exposure. Asbestos use is currently banned in the European Union with the aim to reach a 0 level of exposure. However, it needs to be taken into account that there are still thousands of tons of installed asbestos in the European Union territory.

Secondary prevention is based on an early diagnosis carried out by the units responsible for the surveillance of health of workers exposed to asbestos, and of the exposed general population [18], the establishment of care and rehabilitation measures, and a change of workplace. According to the Protocol of Health Surveillance Specific for Asbestos [19], exposed workers need to follow biannual checkups. Tertiary prevention is the treatment and rehabilitation of the lung function damaged by the ARD.

ARD, particularly the non-malignant forms, is a cause of morbidity and mortality that has been scarcely studied worldwide. Its incidence on the population of the area of this study is unknown.

The latency period between the inhalation of asbestos fibres and ARD is estimated to be around 15 years. It is accepted that the "spontaneous" incidence of mesothelioma is extremely rare, less than 1 new case for one million inhabitants per year [20]. In Western Europe the incidence was around 1 per million until the middle of the 1950s, but currently it has increased to 3 per million per year [6]. No data on the incidence of non-malignant ARD exist.

In Barcelona province the incidence of mesothelioma, estimated from mortality data of residents, is 8.3 cases/million inhabitants/year in men, and 4.7 cases/million inhabitants/year in women, in 1993 [21,22]. More recently [23] the global ARD incidence in the area of study was 9.5 cases/100.000/year. In some towns these figures are much higher: Cerdanyola and Ripollet 35.5 cases/100.000 inhabitants/year. No more data are available and there is a lack of studies oriented to the eventual clinical changes in the ARD follow up.

Incidence data on other ARD in this area are scarce, since it is very rare to register them as a mortality cause, and no prospective studies such as ours exist. The real morbidity and mortality impact of asbestos exposure and the epidemiological and clinical characteristics of the incident cases in this area are not known, but our study is based on the hypothesis that the incidence of non-malignant ARD is also significant.

From 1907 until 1997 one of the larger fibrocement plants in Spain operated in the town of Cerdanyola del Vallès. One of the main components of fibrocement is asbestos. The workers and the general public of this highly populated area have been exposed to asbestos for a long period, and probably they still are due to continued exposure to environmental traces of this material. The identification of increasing numbers of ARD, initially among the fibrocement plant workers but also of other asbestos handling plants, and in the general population in this area of the Barcelona province, has alerted our research team. The current real impact of ARD in this area and the characteristics of the affected people remain unknown.

Of all cases of ARD, an important proportion is related to work exposure. Other ARD cases suggest a non-work or "passive" exposure. This could be just living with or having close contact with a worker that brings asbestos fibres home after finishing the day's work. There are also environmental causes, such as living close to an asbestos source or an industrial asbestos deposit (vicinity). The use of damaged domestic utensils that contain asbestos merits also mention.

In the Brescia province in Italy a register of mesothelioma cases has been established to identify the cases related to work exposure so that they can receive the appropriate benefit [24].

The proportion of cases of mesothelioma attributed to domestic or environmental exposure in the Barcelona province is 26% [15]. For the rest of ARD the percentage attributable to direct work exposure *versus *passive exposure is unknown. None of the studies published to date have include all types of malignant and non-malignant ARD as we aim in our study.

The clinical experience of the study members suggests that most ARD cases are found within the Catalan Health Institute network of primary care and medical specialists, facilitating a comprehensive detection of new cases.

The extended latency period until the onset of symptoms means that even if the disease is mainly work related, most cases will be suspected in the aging population, retired people very often, and in the context of the daily routine of the general practitioner.

Therefore, we propose to carry out a prospective, clinical -epidemiological study based on the comprehensive (all cases), full (all information of every case) and coordinated (among all centres) of the incident cases of ARD between January 2006 and December 2008, with the objective to measure the incidence of ARD in this area, to describe the characteristics of the exposure, and to investigate the space, time and patient profile of the people affected by asbestos.

Previous studies of ARD in Catalonia focus only on mesothelioma incidence, studying mortality. Our group aims at broadening this population-based medical research to the incidence of the other 9 manifestations of ARD to obtain the morbidity and mortality impact of asbestos in the study population.

The active search and investigation of the suspected cases in our area will contribute to its early identification and will raise awareness of this group of diseases among the professionals. Also, they will be able to improve the diagnosis and follow-up of these patients. Concerning health managers and authorities, this information will contribute to plan the future allocation of health resources.

## Hypothesis

The first hypothesis considers that asbestos has played an important role in the industrial development of the study area and therefore it continues to cause a poorly defined and quantified morbidity and mortality by means of a current or past inhalation of asbestos fibres. The exposure can be direct (work exposure) or passive (domestic or environmental) or both.

The second hypothesis states that ARD are underreported. We believe that the notification of work related respiratory diseases caused by asbestos in our area is underreported, and it also affects mortality statistics because of the underreporting of ARD as a cause in death certificates.

The third hypothesis considers the possibility of population clusters of incident ARD cases in the study area that could be compared with one another and that would generate new hypothesis.

## Objectives

### Main objective

The main objective of the study is to measure the incidence rates and the adjusted mortality rates of the 10 illnesses that constitute ARD in the study area. The descriptive mortality and morbidity profile will be adjusted by age and sex, exposure variables and source of exposure (work, environmental or both). Also, the relationship between length of exposure, latency between exposure and first respiratory symptoms, disease diagnosis and decease during the follow up will be studied.

### Specific objectives

-To quantify comprehensively the incident cases and to describe the associated clinical and epidemiological variables.

-To quantify the magnitude of underreporting of ARD in the notified work-related morbidity.

-To quantify the magnitude of ARD underreporting in the death certificates by looking at the mortality statistics of the people whose cause of death was any of the 10 illnesses that constitute ARD.

- The possible detection of geographical clusters of ARD incident cases will contribute to the understanding of the ARD impact on the study population.

## Methods/Design

**1. Study area:** consists of 12 municipalities in the Barcelona province within the catchment area of the health facilities that participate in the study: Cerdanyola del Vallès, Ripollet, Barberà del Vallès, Badia del Vallès, Sabadell, Sant Quirze del Vallès, Santa Perpetua de la Mogoda, Polinyà, Palau de Plegamans-Solità., Castellar del Vallès, Sentmenat and Sant Llorenç Savall. All 12 municipalities refer the patients to the hospital that participates in the project. Nearby towns with a different referral hospital are excluded.

**2. Study population:** anyone who lives in any of the 12 municipalities of the study area and consents to participate in the study. The estimated population is 350,000 inhabitants.

**3. Study network setting:** the study is framed within the area covered by all primary care teams in these 12 municipalities. All these primary care teams belong to one public health provider organization: the Catalan Institute of Health (CIH) and are fully computerised. These teams work with the disease classification of the CIH and refer their patients to the only referral hospital centre in the area, the Consorci Hospitalari Parc Taulí, which belongs to the Public Hospital Network. The only referral Work Health Unit of this area does also participate in the study.

**4. Design: **clinical -epidemiological, longitudinal, prospective study of incident cases diagnosed with any of the 10 diseases that constitute ARD from 1/1/2006 to 31/12/2008.

**5. ARD definition:** ARD is a group of 10 conditions that affect mainly the respiratory system, caused by the inhalation and subsequent deposit of asbestos fibres. According to the guidelines with a broader worldwide consensus [4] ARD are divided in two groups: malignant ARD (pleural mesothelioma, peritoneal mesothelioma, asbestos-related pulmonary carcinoma, and other neoplasms) and non-malignant ARD. The non-malignant ARD comprise diseases of the lung parenchyma (asbestosis or interstitial lung fibrosis), of the pleura (isolated pleural plaques, diffuse pleural fibrosis or pleural thickening and benign pleural effusion), and of the bronchi (chronic bronchial obstruction and round atelectasis).

**6. ARD case definition:** cases are defined as any person within the study area (section 1) first diagnosed with at least one of the ARD (section 5) between 1/1/2006 and 31/12/2008, in accordance with the diagnostic criteria internationally accepted, [4] and who consents to participate in the study. If the patient or his/her family do not sign the informed consent some basic data will still be collected and registered. The basic data for this study are: date of birth, sex, diagnosis and town of current residence. It allows to preserve anonymity and to avoid duplications. All cases with confirmed informed consent that are lost to follow up will be registered and the cause of the loss recorded.

For the diagnosis of ARD, fulfilment of the three following conditions is considered necessary and sufficient: a) either imaging or pathology techniques show that the patient has a lesion compatible with ARD in the respiratory system; b) a sufficient latency period between a work and/or environmental asbestos exposure and/or proof of one or two physiopathological markers of exposure (pleural plaques detected by imaging or presence of asbestos bodies in the bronchoalveolar lavage or in a cytology sample) and c) exclusion of other possible causes of the current disease.

**7. Study variables: ** Appendix 1 describes the study variables. Data are collected by the specifically trained research team by means of a direct interview to the patient or to a close relative.

**8. Circuit of case detection and selection:** awareness and training workshops for the detection of ARD possible cases will be organised for the general practitioners of the study area. The circuit starts when a possible new case is detected. The general practitioner refers it to the respiratory medicine specialist of the area for diagnostic confirmation, then the ICD 10 codes concerning ARD (J61; J92.0; C45) are incorporated in the computerised clinical record of the patient, and a study team physician is notified.

In the primary care teams of Cerdanyola del Vallès, Ripollet, Barberà del Vallès and Badia del Vallès there is a study assigned doctor that will receive the clinical data of the confirmed case from the general practitioner. This assigned doctor is a team researcher trained to collect the variables of the identification file. He/she has an interview with the patient, and if the case fulfils the conditions in sections 1, 2, 3, 5 and 6 the patient is given information about the study (see Appendix 2) and is asked to voluntarily participate. If the patient accepts the informed consent form must be signed (see Appendix 3).

Subsequently the variables obtained by direct interview and through the available clinical information are registered in the identification file. When the file is completed it is sent to the respiratory medicine specialist who centralises and carries out the last quality control of all the study data. This specialist can request additional medical tests and consultations, confirms the diagnosis, checks the identification file, starts the follow-up and refers back the patient to his/her family physician with the clinical information and future management recommendations. The specialist will also send the identification file to the person in charge of entering the data in the single database.

The Primary Care Teams in the towns of Sabadell, Sant Quirze del Vallès, Santa Perpetua de la Mogoda, Polinyà, Palau de Plegamans-Solità, Castellar del Vallès, Sentmenat and Sant Llorenç Savall are also fully computerised, although there is not a doctor allocated to each location because the retrospective study indicates that probably there will be less cases here than in the Cerdanyola-Ripollet area.

Here the detection of cases will take place in primary care, and will be confirmed in the Respiratory Medicine Department of the referral hospital (Consorci Hospitalari Parc Taulí). A study team physician and member of the referral hospital will be in close contact with the Respiratory and Outpatient Departments of the hospital to facilitate the collection of the epidemiological variables of the patients. The epidemiological files will be sent to the ARD specialist, who will centralise and complete the information, and will send them to be included in the project database.

A study team member, respiratory medicine specialist in the Hospital Vall d'Hebron in Barcelona, will identify the cases in the study area that are not captured by the established primary care circuit.

If there are doubts about work exposure in any of the cases, the general practitioner as well as the assigned doctors in the study area can refer it to the referral Work Health Unit, where the doctor in charge will undertake a thorough history and, if needed, will be able to obtain further information on the factories where the patient has worked. The Work Health Unit will send a report to the assigned doctor with the relevant data and the conclusions on the work aetiology of the case.

**9. Collected data entry in a single database:** one of the research team members and the fieldwork assistant will double-entry the variables of the Identification File (see Appendix 1) in the project database to validate the information and for an optimum quality control. A hard-copy of every file will be stored for future checks, together with the informed consent form.

**10. Level of detection of cases: ** an important challenge of the study is the comprehensive detection of incident cases, which will be obtained through the optimization of the circuits described in section 8. For that, the ability of the research team to train and raise awareness among the colleagues in primary care in ARD detection is considered essential. A minimum of two training and reminder sessions will take place with each PCT of the study area just before the beginning of the study, and at least another at the end to present the results and analyse future implications. The Work Health Unit is the referral point of all PCT in the area for the work related pathology, and it actively participates in the sensitization of the family doctors to detect and notify suspected ARD cases.

The Consorci Hospitalari Parc Taulí is the referral hospital for the whole study area, and besides furthering the study of the ARD cases for diagnostic confirmation, new ARD cases can be detected in its Emergency or Respiratory Medicine Outpatient Departments. The fieldworker will undertake every three months a comprehensive search for every computerised new case of primary care and hospital discharge reports with the relevant ICD 10 codes (J61; J92.0; C45). A member of the research team will support the fieldworker to ensure the uniformity and quality of the information.

**11. Estimate of the detected new cases figures:** this is a population based study, of which of the 10 diseases that constitute ARD, we only know the incidence rate of mesothelioma for Barcelona province. Due to the lack of known rates for the other ARD, the final size calculation was based on the actual frequency of ARD new cases in primary care in the study area. We consider that it is very difficult to estimate the total number of incident cases that we will find, but we are confident that the three years' duration of the study will allow us to obtain the incidence of all forms of ARD in the area. However, based on the approximate incidence of mesothelioma in this area, we would expect to find a mean of 6 cases of mesothelioma per year. The number of non malignant ARD diseases should be higher, and may not yet have prompted the patient to seek medical care.

**12. Statistical Analysis:** a descriptive and inferential statistics of the variables in the database will be carried out for the analysis of the information. The exposure profile and the space-time-patient variables of the patients will be determined. The incidence and mortality rates adjusted by age, sex and ARD form will be calculated. The respective Mantel-Haenszel Odds Ratio for the confounder variables will be determined, and logistic regression and survival analysis will be used to complete the study. The significance level was set at 5%.

The estimate of the underreporting in the cause of death certification in the mortality statistics of the Catalan Government mortality register will be carried out based on the analysis of the death bulletins of the cases that have died during the follow up due to any of the ARD conditions.

The potential existence of population clusters of cases in the town of Cerdanyola del Vallès will be studied, since in this municipality there was an asbestos related factory until the end of the 1990s. The geographical areas for cluster analysis will range from 100 to 500 metres, though this measure will be reviewed in relation to subsequent inter-case proximity criteria.

**13. Ethics:** when the first direct contact of the study investigator with the patient (or his/her family in case of death previous to the first contact with a research physician or due to the absence or incapacity of the patient to answer himself) is established, the project is explained and the voluntary participation in the study requested. Also, the objective and characteristics of the study (Appendix 2) are explained in detail and the signature of the informed consent form is requested (Appendix 3). The informed consent form stipulates the basic ethical considerations with regard to the right to privacy, anonymity, confidentiality, cancellation and information. The research team agrees to follow the ethical rules of the accepted practice established in the deontological code of the Barcelona Official College of Physicians valid since 20/11/2004.

The study has been favourably evaluated by the Research Committee of the IDIAP Jordi Gol.

## Discussion

The main limitation of the study is that ARD is nor a common descriptor of reported morbidity, nor a common basic cause of certified death. Also, ARD are underreported as work related diseases. Finally, the quality of the available information can be limited by the long latency period between exposure and disease, the lack of an exact measure of asbestos exposure and the memory bias of the patients and/or relatives, and the different degree of quality in the information obtained for each case depending on the different sources consulted.

To overcome these limitations, the research team aims to awake the awareness of general practitioners to notify cases. To check duplicities, each case will be matched against all data bank. Likewise, during the study period a quality control of the notification with the data of the Hospital Discharge Report (CMBD) will be established.

## Conclusions

The results and conclusions of the study will allow for a broad range of scientific and academic health and non-health related activities to unfold within the framework of health promotion and safety and of primary, secondary and tertiary disease prevention. Similarly, the experience and information obtained will allow the research team to spread this information through scientific papers and training activities for different technical, managerial and organisation groups, and to open the way to a future unified register of ARD.

It is a unique opportunity for the institutions represented by the members of the research team to increase the value of its investigation in the areas of health care and applied medical research. If the understanding of ARD, its incidence and prevalence are better known, higher quality information and data will be followed by a broader clinical consensus. This in turn will allow for the development and diffusion of specific clinical guidelines for ARD within the framework of quality healthcare management of primary care. The proposal to implement ARD Specific Follow-up Units will be encouraged, and the current scientific literature on ARD will increase.

The conclusions of the current study can contribute to better reporting practices of ARD as a work related condition. This would have social, financial and care benefits for the patients and their families. In general, it will contribute to minimise the risk of acquiring ARD also by advising on the hazardous use of poorly preserved domestic asbestos-containing tools.

## Abbreviations

ARD: Asbestos Related Diseases; PCT: Primary Care Team; IDIAP: Primary Care Research Institute; CIH: Catalan Institute of Health.

## Competing interests

The authors declare that they have no competing interests.

## Authors' contributions

MR, RA and JT are the main investigators, responsible for the idea of the project and the writing of the manuscript. JT is the respiratory medicine consultant for the study with a broad experience in ARD. He decides which cases meet the diagnostic criteria and are included in the study. JT is also in charge of training the fieldworker, and analyses and suggests improvements in the flow of study patients. IG, EC, IK and XM contribute to the recruitment and identification of asbestos possible cases in the primary health centres of the study. XM is responsible for creating the database, data entry and quality control. GC and MG coordinate the cases detected in the different departments of the referral hospital, and refer the patients to the respiratory medicine consultant for confirmation. CA is responsible for the cases with a suspected work exposure, and for defining the source of asbestos exposure in each case. All authors have read and approved the final version of the manuscript.

## Appendix 1

IDENTIFICATION CODE      **            **      **DATE REGISTERED**

(1) PATIENT ID

(2) CASE NUMBER:

(3) ORIGIN:

1: HOSPITAL

2: PRIMARY CARE HEALTH CENTRE

3: PRIMARY CARE MANAGEMENT

4: PRIVATE INSURANCE COMPANY

5: HEALTH WORK DEPARTMENT

6: PRIVATE

7: OTHER

(4) ORIGIN DESCRIPTION:

(5) PRIMARY CARE CLINICAL RECORD NUMBER:

**(6) SEX: **0: MAN; 1: WOMAN

(7) DATE OF BIRTH:

**(8) PLACE OF BIRTH: **TOWN:      **      **    PROVINCE:      **      **    COUNTRY:

(9) TOWN OF RESIDENCE:

(10) SOURCE OF EXPOSURE:

1.-CERDANYOLA ASBESTOS CEMENT PLANT

2.-OTHER PLANTS (SPECIFY):

3.-VICINITY (SPECIFY)

4.-FAMILY (SPECIFY):

5.-OTHER (SPECIFY):

9.-UNKNOWN

(11) EXPOSURE INTERVAL:

BEGINNING EXPOSURE INTERVAL 1:_____   FINAL EXPOSURE INTERVAL 1:_____

BEGINNING EXPOSURE INTERVAL 2:_____   FINAL EXPOSURE INTERVAL 2:_____

(12) SMOKER

0.-NO

1.-YES____   Number Cigarettes/Day:____   During (Years):____

2.-EX-SMOKER____   Number Cigarettes/Day:____   During (Years):____

9.-UNKNOWN

(13) DIAGNOSIS DATE:

**(14) CONSIDERED WORK-RELATED DISEASE: **(0 = NO, 1 = YES, 9 = UNKNOWN)

(15) SYMPTOMS:

1.-ASYMPTOMATIC

2.-PERSISTENT DRY COUGH

3.-RESPIRATORY FAILURE

4.-CANCER SYMPTOMS

9.-OTHER, SPECIFY:

**(16) PHYSICAL EXAMINATION: **CLUBBING CRACKLES

(0 = NO, 1 = YES, 9 = UNKNOWN)

(17) IMAGING

1.- X-RAY

2.- CT SCAN

3.- CT SCAN -LUNGS

4.- MRI

5.- ULTRASOUND

6.-OTHER

(18) IMAGES

1.- PLEURAL PLAQUES

2.- PLEURAL THICKENING

3.- ROUNDED ATELECTASIS

4.- PULMONARY FIBROSIS

5.- PARTIAL PLEURAL EFFUSION

6.- MASSIVE PLEURAL EFFUSION.

7.- OTHER (SPECIFY)

(19) PATHOLOGY TECHNIQUES:

1.- BIOPSY

2.- AUTOPSY

3.- CYTOLOGY

4.- OTHER

9.- UNKNOWN

(20) PATHOLOGY RESULT:

**(21) LFT:____   FVC**:____%    **FEV1**:____%   **Tiffenau Index:____**%   **TLCO**:____   %, **DATE:____**

(22) CLINICAL PROGRESSION

1.-STABLE

2.-DETERIORATION

3.-DEATH

9.-UNKNOWN

(23) DATE OF DEATH

## Appendix 2

INFORMATION LEAFLET FOR THE STUDY PARTICIPANTS

PROSPECTIVE STUDY OF INCIDENCE CASES OF ASBESTOS RELATED DISEASES IN PRIMARY CARE IN A HEALTH AREA OF BARCELONA PROVINCE

### Study objective

To know the clinical and epidemiological features of the people affected by asbestos exposure.

### Study procedures

You have clinical features that can be a consequence of asbestos exposure. If you accept to participate in the study, you will be assigned to a study doctor who will carry out a clinical and work history interview and a specific clinical examination to determine if you might be affected by asbestos exposure. You might be asked to provide clinical records and/or diagnostic tests carried out in the past, to determine when the disease might have started. It is possible that the doctor requests additional diagnostic tests to confirm the diagnosis.

At the end of this process you will be informed on whether you are or not affected by ARD, and if you should take any measure to improve your health.

Your participation in the study is voluntary, and at any point you can withdraw from it. If you withdraw there will not be any consequences regarding your clinical care.

The data collected during the study will be treated as confidential. In the study lists only your allocated study number will appear. In the final report of the study, or in the case of communicating these results to the scientific community, your identity will remain anonymous. Information will be provided, as is anticipated in the article 5 of the Organic Law 5/1992 of Automated Treatment of Personal Data regulation, regarding the automated treatment of these data, and of the rights of the participants to consult, modify or delete from the file their personal data.

This study does not provide with any financial compensation, not even the cost of transport to the Health Centre.

**Study researchers: **if you have any doubt concerning this study, or you would like to comment on some aspect of it, you could phone............................or get in touch with your general practitioner.

Once you have read this information and clarified possible doubts, if you want to take part in the study you will have to sign the informed consent form.

## Appendix 3

INFORMED CONSENT FORM

PROSPECTIVE STUDY OF INCIDENCE CASES OF ASBESTOS RELATED DISEASES IN PRIMARY CARE IN A HEALTH AREA OF BARCELONA PROVINCE

I.....................................................................................................(name and surname)

I have read the information leaflet

I have been able to ask questions about the study

I have received enough information on the study

I have spoken to ..................................................(name of the attending GP)

I understand that my participation is voluntary

I understand that I can withdraw from the study:

1.- When I want

2.- Without having to explain

3.- And it will not have any consequences on my medical care

I freely consent to participate in the study today,

In............................................................day..............month.............................. 20......

..................................................................................   .......................   .................

Name and surnames of the participant                     Signature         Date

................................................................................   ........................   ....................

Name and surnames of the attending GP                        Signature         Date

## Pre-publication history

The pre-publication history for this paper can be accessed here:

http://www.biomedcentral.com/1471-2458/10/203/prepub
